# Validation of factor structures of the Drinking Motives Questionnaire among the Czech young and adult general population

**DOI:** 10.1186/s40359-024-02002-2

**Published:** 2024-09-27

**Authors:** Ladislav Kážmér, Ladislav Csémy, Ondřej Šíba

**Affiliations:** National Institute of Mental Health, Topolová 748, Klecany, 25067 Czech Republic

**Keywords:** Drinking motives, Alcohol use, General population, Factor analysis, Czech Republic

## Abstract

**Background:**

Alcohol use is one of the leading public health concerns in the Czech Republic. Drinking motives play a vital role in both initiation and subsequent alcohol use. A revised version of the self-report Drinking Motives Questionnaire (DMQ-R) has been proposed to assess these motives. The present study aims to validate the DMQ-R in the Czech general population.

**Methods:**

A total sample of 1,784 Czech participants completed a national survey. For the analysis, only a sub-sample of the past 12 months alcohol users was used: *N* = 1,123; 52.8% male; mean (SD) age = 40.2 (13.3). Drinking motives were assessed by the adopted Czech version of the DMQ-R. Both confirmatory (CFA) and exploratory factor analysis (EFA) were conducted to examine the factorial structure of the instrument. The age of the participant was additionally considered in the analysis (15–24 years as opposed to 25–64 years).

**Results:**

The CFA supported the four-factor model in the 25–64 age group. The analysis supported the construct validity of the Social, Conformity, and Coping factors. The Enhancement factor retained only two items and was found to refer more to a domain of ‘Pleasant Feeling’. For the 15–24 age group, the hypothesised four-factor structure was not corroborated.

**Conclusions:**

The Czech version of the DMQ-R was found to be a reliable measurement tool of the Social, Conformity, and Coping motives. Future research should investigate the dimensionality of the instrument items presumed to correspond to the Enhancement motives. This should be conducted particularly among adolescents and young adults aged 15–24 years, where administering the DMQ-R with a large enough sample is also needed.

**Supplementary Information:**

The online version contains supplementary material available at 10.1186/s40359-024-02002-2.

## Background

Excessive alcohol use is a significant public health concern, associated with a range of cardiovascular diseases, several types of cancer, deteriorating mental health and other alcohol-related problems [1–3]. According to the World Health Organization [4], harmful alcohol use contributes to an estimated 3 million deaths worldwide each year. To reduce the harms associated with alcohol consumption, prevention must be based not only on an understanding of the prevalence and patterns of alcohol use, but also on a thorough understanding of the motives that act as a catalyst for this behaviour [5–7]. These drinking motives are defined as personal needs that people seek to satisfy through the alcohol use [8, 9].

The Cox & Clinger’s [8] motivational model suggests that the ultimate pathway to alcohol use is the desire to achieve affective change through drinking. To better understand an individual’s motivation to drink, the original Drinking Motives Questionnaire (DMQ) was developed by Cooper et al. [10]. The main purpose of the DMQ was to measure the extent to which people drink either to enhance a positive outcome or to avoid a negative one. The original DMQ consisted of 15 items measuring three underlying motivational components – social (to achieve social benefits), coping (to relieve negative mood states), and enhancement (to improve positive mood states).

Based on the DMQ, Cooper [11] later developed a revised version referred to as DMQ-R, adding a fourth motivational factor – conformity (to avoid social rejection). She included these motives based on the assumption that adolescents, as well as adults who are susceptible to social pressure, may consume alcohol in order to conform to the drinking expectations of their social group. This inclusion led to the development of a comprehensive four-factor questionnaire designed to assess each of the motives simultaneously. Over the past few decades, the DMQ-R has become a leading instrument for measuring of drinking motives and has been used worldwide [5].

In the DMQ-R, Cooper [11] suggests that social motives receive positive reinforcement and are externally focused, e.g., drinking to celebrate a special occasion with a friend. In contrast, conformity motives involve negative reinforcement and an external focus, e.g., drinking, so ‘you won’t feel left out’. Coping motives are negatively reinforced with an internal focus, e.g., drinking ‘to forget worries’. Conversely, enhancement motives receive positive reinforcement but are internally focused, e.g., drinking because ‘it gives you a pleasant feeling’.

The construct validity and psychometric properties of the DMQ-R have been most extensively tested in adolescent populations, including the United States [11, 12], Hungary and Spain [13], as well as Switzerland [14] and Italy [15]. However, the original motivational model of alcohol use by Cox & Klinger [8] was considered more general and not limited to the adolescents. Therefore, some studies have also examined the DMQ-R in the adult population [16–18]. Recently, Crutzen & Kuntsche [16] tested the factor structure of the DMQ-R on a representative sample of the adult population in the Netherlands and concluded that the four-factor structure of the DMQ-R is also appropriate for measuring drinking motives in adults.

Since alcohol use is one of the leading public health concerns in the Czech Republic [19, 20], the present study aims to examine the latent factorial structures of drinking motives in the Czech general population using the adopted version of the DMQ-R questionnaire. To the best of the authors’ knowledge, the psychometric properties of the DMQ-R have not been examined in the Czech cultural context, especially not in a large-scale, nationally representative survey. In addition to the validity of the factorial structures of the DMQ-R instrument and its presumed practical use within the Czech population, the examination of latent motivational structures for alcohol use may also contribute to the basic question of whether there are some specifics relevant to the Czech drinking culture.

The study presented in this paper is structured as follows. The underlying theoretical foundations of the DMQ-R are discussed in order to provide the conceptual background for testing the factor structures of the questionnaire in the Czech population. Subsequently, in the first major step of the analysis, the factor structures are tested on the general Czech population aged 15–64. In the light of the empirical results obtained for the general population, these structures are examined in more detail in the second major step of the analysis. Specifically, two age subgroups of the general population are considered separately: adolescents and young adults (15–24 years) as opposed to adults (25–64 years). As the drinking motives of adolescents and young adults are closely related to the socialisation processes of the maturing population, in which alcohol traditionally plays a facilitating role, the analytical distinction is applied particularly to the younger subgroup. The final part of the paper discusses the specific factorial structures identified for the two respective age subgroups. Implications for future research in the Czech national context are also provided.

## Material and methods

### Sample and procedure

The administration of the DMQ-R questionnaire was part of the annual National Survey on Tobacco and Alcohol Use in the Czech Republic, conducted by the National Institute of Public Health [20]. The sampling of respondents for the survey was a two-stage process. In the first stage, 211 electoral districts were randomly selected from a complete list of electoral districts in the country. In the second stage, trained interviewers sought respondents within a selected electoral district using a random walk with a quota table. A total of 1,987 persons aged 15 years and over were approached in the second stage, of whom 1,784 agreed to participate in the national survey (response rate 89.8%). The sample was representative of the country’s population in terms of gender, age groups in five-year cohorts and all 14 administrative regions of the Czech Republic. Data collection took place between 21st November and 6th December 2022. Trained interviewers conducted a structured face-to-face survey interview about the participants’ tobacco and alcohol use and then asked them to complete the DMQ-R self-report questionnaire. The entire survey took about 45 min to complete.

The target population of the study was the general Czech population of working age^1^, aged 15–64. To allow for an age-specific approach, this target group was further divided into two age subgroups (15–24 and 25–64). Only those respondents who reported any alcohol use in the past 12 months were included in the study, i.e. current abstainers were excluded. In total, the final sample we worked with consisted of 1,123 individuals, 52.8% male, with a mean age of 40.2 years (SD 13.3).

### Ethical considerations

Participation in the survey was voluntary for all respondents. Prior to the interview, respondents were fully informed about the purpose of the survey, including statements about anonymity, confidentiality and discretion of data use.

Participants gave their verbal consent to participate in the study. In the Czech Republic, signed informed consent to participate is only required for clinical trials. For anonymous population surveys, only full information about the purpose of the survey and voluntary participation is required. For more information, see Declarations section.

### Instrument and its theoretical framework

The main aim of the study was to examine the factorial structure of the Drinking Motives Questionnaire-Revised [11]. The DMQ-R consists of 20 items rated on a 5-point Likert scale: (i) Almost never/Never, (ii) Some of the time, (iii) Half of the time, (iv) Most of the time, (v) Almost always/Always. The questionnaire was adapted into Czech using a standard procedure. Namely, two independent translations into Czech (LCs and LK) were made. In order to check the functional equivalence of the translation, the final Czech version was back-translated into English by a bilingual collaborator.

**Fig. 1:**
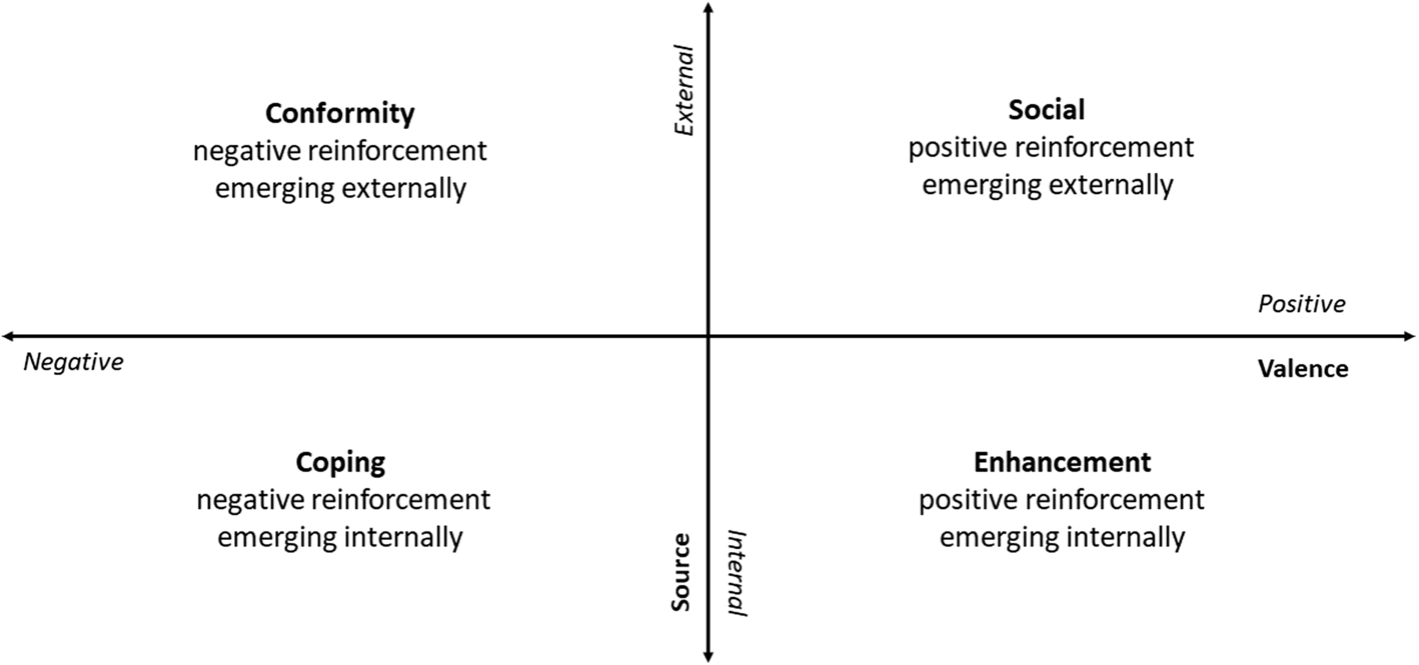
Theoretical dimensions and domains of the DMQ-R

In developing the analytic strategy, we reflected on the theoretical foundations on which the DMQ-R was built. Cooper [11] drew on the motivational model of alcohol use, which posits that motivations to drink alcohol can be captured along two main dimensions. The first, *valence*, reflects the positive or negative expectations associated with drinking; the second dimension reflects whether the *source* of the expectation of drinking effects is external or internal. Thus, in this theoretical framework, an individual drinks alcohol to obtain its positive effects (positive reinforcement) or, conversely, to avoid its negative effects (negative reinforcement). Similarly, drinking may be motivated to induce a desirable internal emotional state (internal rewards) or to gain social acceptance or approval (external rewards). The four-factor model of drinking motives in the DMQ-R represents the four domains created by the intersection of these dimensions, as shown graphically in Fig. 1. The four factors are *enhancement* – internal motives emerging as positive reinforcement, *social motives* – positive reinforcement emerging externally, *coping* – internally emerging negative reinforcement, and *conformity* – negative reinforcement emerging externally.

The theoretical underpinnings were incorporated into the analytical strategy of the data analysis. This included testing for alternative factor structures following the procedure suggested in Cooper [11]. Specifically, the statistical fit of the following set of factor structures was consecutively tested: (1) an initial one-factor solution with all items collapsed together; (2) a two-factor structure of internal vs. external drinking motives (hereafter referred to as Two-factor A); (3) a two-factor structure of positive vs. negative motives (referred to as Two-factor B); (4) a three-factor structure of positive reinforcement (with social motives and enhancement combined) vs. factors of coping, and conformity; (5) a four-factor structure distinguishing between all the hypothesised latent domains.

### Statistical analysis

The analytical strategy involved several consecutive steps of statistical analysis and data modelling. The aim was to provide a detailed examination of the underlying latent structures of the DMQ-R items, considering both the exploratory and confirmatory analytical frameworks.

The confirmatory framework is represented here by the set of confirmatory factor analysis (CFA) models, conducted in the first and the last major steps of the analysis. In the first major step, the pre-defined latent structures were formally tested through the set of consecutive CFA models. As the tests did not corroborate any of the hypothesised factor structures at the conventionally desired level of the model fit statistics^2^, the exploratory factor analysis (EFA) was carried out in the second major step of the data analysis. Based on the results of the EFA, the final CFA model with a reduced number of DMQ-R items was derived and formally tested in the third major step. For this final CFA model, the reliability and discriminant validity were assessed using the commonly used indices used for this purpose – the Cronbach’s α, the McDonald’s ω, the average variance extracted (AVE) and its square root (√AVE).

Given the ordinal nature of the DMQ-R responses, specialised methods developed for advanced multivariate analysis of Likert-type data were applied in all steps of the analysis. Specifically, the latent ordinal response CFA models based on polychoric inter-item correlations, as defined by Muthén [21] and later recommended by a number of other methodological studies^3^ [22–24], were applied in the first and last steps of the data analysis. The parameter estimates of the CFA models were obtained using the robust diagonally-weighted least squares (DWLS) estimation procedure with mean-and variance-corrected test statistics and robust standard errors. In such CFA models, the Likert-scale dependent variables (i.e. the input DMQ-R items) are treated as manifest ordinal values of their latent continuous response variables operating in the background. In order to analyse the covariance structures of these latent response variables, polychoric correlations between the observed input items are first estimated. Then, the thresholds of the latent CFA responses are empirically derived from the cumulative distribution of the observed ordinal items. Both the polychoric correlations and the thresholds are then entered into the latent CFA model. For the estimation of CFA model parameters, the DWLS method is preferred over other estimation procedures [25]; hence the approach applied in our analyses. For a more detailed discussion in this regard, see e.g. Muthén [21] and Kline [24].

Regarding to the covariances between the extracted CFA latent factors, we note that the latent factors were allowed to correlate freely. However, no other covariances except of these inter-factor ones were assumed, due to the lack of theoretical justification for such an assumption; thus, the CFA models did not allow for any covariance structure between the residual terms.

For the EFA conducted in the second major step of the data analysis, we proceeded to divide the respondents into two subgroups defined by their age: a) the subgroup of adolescents and young adults aged 15–24 years, and b) adults aged 25–64 years. In this second step, the factorial structures of the DMQ-R were thus explored separately in the two age subgroups.

We also emphasise that the complex major steps of data analysis described above were preceded by an initial statistical examination of the input DMQ-R items. This was both in terms of exploring the basic distributional properties of the data (skewness, kurtosis) and checking their statistical suitability for factor analysis as a specific multivariate method of data modelling (examining the communalities and squared multiple correlations of the input items, calculating the Kaiser–Meyer–Olkin measure of sampling adequacy, checking for possible collinearity between any of the items in terms of examining the set of eigenvalues and the value of the discriminant).

For the sake of technical completeness of all the procedures carried out in the analysis, we also refer to the following statistical packages that were applied. For the latent ordinal response CFA models, the ‘lavaan’ package [26] for the R Statistical Software [27] was used, applying the recent syntax provided by Kline [24]. Reliability indices for the final CFA model were computed in the ‘semTools’ package [28]. The analysis of the EFA structures was performed in the Stata-15 statistical software [29], using the ‘polychoric’ command to compute the polychoric correlation matrix and the ‘factor’ command to extract the EFA factors.

## Results

Table 1 provides an overview of all 20 DMQ-R items used in the analyses and their basic descriptive statistics. The original English version of the items and their Czech translation are presented. The correspondence of each item to the theoretical domain (latent factor), as implied by the theoretical framework of the DMQ-R instrument, is also presented.

**Table 1:** Descriptive statistics of the input data

**Questionnaire item**	**English version** (Cooper, 1994)	**Item translated into the Czech**	**(Domain)**	**Min**–**Max**	**Mean**	**Std. Dev**
DMQ1	To forget your worries	Abyste zapomněl/a na starosti	(Coping)	1–5	1.84	1.08
DMQ2	Because your friends pressure you to drink	Protože vás k pití nabádají přátelé	(Conformity)	1–5	1.89	1.09
DMQ3	Because it helps you enjoy a party	Protože si líp užijete párty	(Social)	1–5	2.29	1.27
DMQ4	Because it helps you when you feel depressed or nervous	Protože vám to pomáhá, když máte pocity deprese nebo nervozity	(Coping)	1–5	1.76	1.09
DMQ5	To be sociable	Abyste byl/a společenštější	(Social)	1–5	2.03	1.19
DMQ6	To cheer up when you are in a bad mood	Abyste se rozveselil/a, když máte špatnou náladu	(Coping)	1–5	1.90	1.16
DMQ7	Because you like the feeling	Protože máte rád/a pocit, který to vyvolává	(Enhancement)	1–5	1.83	1.17
DMQ8	So that others won't kid you about not drinking	Aby si z vás ostatní neutahovali, že nepijete	(Conformity)	1–5	1.37	0.87
DMQ9	Because it's exciting	Protože je to vzrušující	(Enhancement)	1–5	1.52	0.99
DMQ10	To get high	Abyste si udělal/a náladičku	(Enhancement)	1–5	2.20	1.25
DMQ11	Because it makes social gatherings more fun	Protože to dělá společenská setkání zábavnějšími	(Social)	1–5	2.24	1.24
DMQ12	To fit in with a group you like	Abyste zapadl/a do skupiny, kterou máte rád/a	(Conformity)	1–5	1.61	1.01
DMQ13	Because it gives you a pleasant feeling	Protože to ve vás vyvolává příjemné pocity	(Enhancement)	1–5	1.99	1.18
DMQ14	Because it improves parties and celebrations	Protože to zlepšuje oslavy a párty	(Social)	1–5	2.35	1.31
DMQ15	Because you feel more self-confident and sure of yourself	Protože pak cítíte větší sebedůvěru a sebejistotu	(Coping)	1–5	1.87	1.15
DMQ16	To celebrate a special occasion with friends	Abyste oslavil/a s přáteli zvláštní příležitost nebo událost	(Social)	1–5	2.99	1.43
DMQ17	To forget about your problems	Abyste zapomněl/a na své problémy	(Coping)	1–5	1.77	1.13
DMQ18	Because it's fun	Protože je to zábavné	(Enhancement)	1–5	1.93	1.20
DMQ19	To be liked	Abyste se líbil/a	(Conformity)	1–5	1.35	0.85
DMQ20	So you won't feel left out	Abyste neměl/a pocit vyřazenosti	(Conformity)	1–5	1.37	0.83

Past 12 months alcohol users, age 15–64 years only. Czech adult population sample (2022), sample size *N* = 1,123

The descriptive statistics in Table 1 are evaluated within the sample of the Czech respondents who reported i) having consumed alcohol in the last 12 months prior to the date of the survey and ii) being in the age range of 15–64 years. In total, *N* = 1,123 respondents met both of these conditions. Although not explicitly presented in the table of descriptive statistics, we note that the distribution of several 5-point Likert-scaled items was also characterised by a significant asymmetry (with skewness > 2) combined with a leptokurtotic distribution (kurtosis > 7). This was particularly the case for items DMQ8, DMQ19 and DMQ20.

Table 2 presents the results of the initial CFA analysis aimed at testing for alternative factor structures of the full 20-item set of the DMQ-R in the Czech general population aged 15–64 years. Specifically, CFA model fit statistics are presented in a comprehensive manner, testing for factor structures progressively from a most parsimonious (one-factor) latent structure, through two-, three-, up to the four-factor CFA model. Both exact (χ^2^, scaled χ^2^) and approximate model fit statistics (RMSEA, CFI, TLI, SRMR) are presented.

**Table 2:** Comparison of alternative factor structures, goodness of fit statistics

Number of factors:	One	Two^1)^ A	Two^1)^ B	Three^1)^	Four^1)^
Latent factor structure:	Collapsed	Internal *vs.* External motives^a)^	Positive *vs.* Negative motives^b)^	Positive reinforcement^c)^*vs*. Coping, and Conformity	Social, Coping, Conformity, and Enhancement
N	1 123	1 123	1 123	1 123	1 123
Model χ^2^	3 538.8	2 923.5	2 459.3	1 773.0	1 527.3
*Df*	170	169	169	167	164
[Scaling correction factor]^2)^	[0.950]	[0.920]	[0.911]	[0.748]	[0.699]
Model scaled χ^2^	3 805.0	3 258.3	2 778.8	2 435.8	2 246.4
RMSEA	0.138	0.128	0.117	0.110	0.106
(90% CI RMSEA)	(0.134–0.142)	(0.124–0.131)	(0.114–0.121)	(0.106–0.114)	(0.102–0.110)
CFI	0.881	0.899	0.915	0.926	0.932
TLI	0.867	0.887	0.904	0.916	0.921
SRMR	0.104	0.098	0.087	0.073	0.068

Past 12 months alcohol users, age 15–64 years only, Czech adult population sample (2022)

The alternative factor structures defined as latent ordinal response variables CFA models, based on polychoric inter-item correlations with observed thresholds. Estimation method – diagonally-weighted least squares (DWLS), mean-and variance-corrected

*RMSEA* Root mean squared error of approximation, *CFI* Comparative fit index, *TLI* Tucker-Lewis index, *SRMR* Standardised root mean squared residual

^1)^ The latent factors were allowed to correlate freely

^2)^ Scaling correction factor for the scaled χ^2^ statistic. The scaled χ^2^ was used to calculate the approximate model fit statistics (RMSEA through SRMR)

^a)^ Internal motives: Coping + Enhancement; External motives: Social + Conformity

^b)^ Positive motives: Social + Enhancement; Negative motives: Coping + Conformity

^c)^ Positive reinforcement: Social + Enhancement

Comparing the fit statistics in Table 2, the four-factor structure appears to be more appropriate than the other models with fewer latent factors. This is particularly the case when comparing the four-factor model with either the one-factor (collapsed) or any of the two-factor models (Two-factor A and/or Two-factor B). Nevertheless, the comparison of the four-factor model with the more parsimonious three-factor structure yields similar results: a substantial χ^2^ difference value of ~ 200 with only *df* = 3 restricted degrees of freedom, thus favouring the four-factor model in a highly significant way (*p* < 0.001).

The results of the initial CFA tentatively corroborated the four-factor structure of the full 20-item DMQ-R over of the simpler structures. However, none of the initial models achieved the desired level of acceptability, as documented by the fit statistics in Table 2. Therefore, a more detailed examination of the data was undertaken in the second major step of the analyses.

Table 3 provides the results of the exploratory factor analysis (EFA) of the DMQ-R with a special focus on the two subgroups of respondents defined by age – adolescents and young adults (aged 15–24 years) and adults (aged 25–64 years). Within each subgroup, four principal factors with the largest eigenvalues were extracted from the polychoric inter-item correlations, which were then rotated (oblique oblimin rotation). The standardised factor loadings are presented in Table 3; the DMQ-R items are sorted to create clusters of variables that load on the factor to which they are expected to correspond (see the hypothesised correspondence to latent domains presented in Table 1). Data on item communality (h^2^) and item complexity are also reported.

**Table 3:** Exploratory factor analysis (EFA), by two age subgroups

**Item**	**Age group: 15–24 yrs.** (n_1_ = 163)						**Item**	**Age group: 25–64 yrs.** (n_2_ = 960)					
	Standardised factor loading				h^2^	compl		Standardised factor loading				h^2^	compl
	F1	F2	F3	F4				F1	F2	F3	F4		
DMQ3	0.881			0.260	0.79	1.19	DMQ3^a)^	0.820				0.71	1.03
DMQ5	0.632		0.274		0.67	1.38	DMQ5	0.592	0.297			0.72	1.52
DMQ11	0.827				0.65	1.11	DMQ11^a)^	0.766				0.73	1.03
DMQ14	0.972				0.79	1.04	DMQ14^a)^	0.814				0.77	1.10
DMQ16	0.823		-0.359		0.54	1.49	DMQ16^a)^	0.707				0.47	1.22
DMQ2			0.356	0.845	0.92	1.47	DMQ2	0.393	0.479			0.55	2.20
DMQ8	-0.250		0.994	0.323	0.92	1.34	DMQ8^a)^		0.988			0.85	1.01
DMQ12			0.876		0.76	1.11	DMQ12^a)^		0.773			0.77	1.13
DMQ19	0.227		0.698		0.80	1.28	DMQ19^a)^		0.746	0.261		0.91	1.26
DMQ20			0.913		0.87	1.02	DMQ20^a)^		0.856			0.87	1.06
DMQ1		0.999			0.83	1.07	DMQ1^a)^				0.932	0.76	1.01
DMQ4		0.804	0.282		0.83	1.30	DMQ4^a)^				0.819	0.76	1.07
DMQ6	0.247	0.713			0.77	1.26	DMQ6	0.342			0.467	0.72	2.11
DMQ15	0.416	0.379		-0.289	0.72	3.03	DMQ15	0.235	0.222		0.319	0.61	3.28
DMQ17		0.934			0.84	1.02	DMQ17^a)^				0.790	0.78	1.04
DMQ7	0.669				0.69	1.26	DMQ7^a)^			0.626		0.78	1.27
DMQ9	0.422		0.505		0.66	1.95	DMQ9		0.434	0.510		0.77	1.97
DMQ10	0.792				0.69	1.01	DMQ10	0.578		0.371		0.77	1.79
DMQ13	0.661				0.57	1.10	DMQ13^a)^			0.724		0.81	1.16
DMQ18	0.918				0.84	1.03	DMQ18	0.312		0.606		0.80	1.77

Past 12 months alcohol users only, Czech adult population sample (2022)

Extraction method – principal factors, based on polychoric inter-item correlations; oblique rotation – oblimin

h^2^ – communality; compl – Hoffman’s index of item complexity (Hofmann, 1978) (Index of item complexity – the number of common factors that an item is involved in its factorial description [30]; for a simple structure, low values of the index are desirable.)

Standardised loadings with an absolute value less than 0.200 are suppressed

^a)^ Subsequently used for the CFA conducted on the reduced set of DMQ-R items

There are several important findings in Table 3. First, the hypothesised four-factor structure is not corroborated within the younger 15–24 subgroup. Although some questionnaire items have a substantial loading on the extracted F4 (e.g. DMQ2), these items are far from those that were hypothesised to load on the extracted common factor (i.e. from the items for a domain of Enhancement). Contrary to expectations, the items for the Enhancement domain load rather on the first extracted factor F1 (i.e. the factor corresponding to Social motives); this is particularly the case for DMQ7, DMQ10, DMQ13, and DMQ18. Thus, for the younger subgroup, the drinking motives that were originally hypothesised to be Enhancement-driven appear to be more related to the Socially-driven factor.

Second, within the subgroup of adults aged 25–64, the EFA revealed several DMQ-R items characterised by substantial loading on multiple extracted factors. Looking at the factor loadings presented in Table 3, this was particularly the case for items DMQ2, DMQ9, DMQ10, as well as DMQ6 and DMQ15. These items were therefore excluded from further analysis.

Furthermore, there were some DMQ-R items with a high loading on the main factor they were hypothesised to correspond, but also with a relatively substantial cross-loading on some secondary factor (resulting in a rather undesirable item-complexity above 1.5, see items DMQ5 and DMQ18).

As a result of these EFA structures, only those DMQ-R items that met the following two conditions were retained for the final step of the analysis: i) factor loading on the corresponding main EFA factor > 0.700 (indicating that ~ 50% or more of an item’s variability is devoted to the extraction of this latent factor), and ii) eventual cross-loading of an item on any other secondary factor < 0.300. The DMQ-R items that meet these two conditions are marked in Table 3.

Given the discrepancy between the hypothesised and data-implied factor structure within the younger 15–24 subgroup, only the subgroup of adult respondents aged 25–64 was considered in the last step of the analysis. The aim here was to test, within the framework of the CFA, the four-factor structure on the reduced set of DMQ-R items (set of 13 items retained). The results of this final CFA are presented in Table 4.

**Table 4:** Confirmatory factor analysis (CFA) on the reduced set of DMQ-R items, four-factor latent structure

Questionnaire item	Latent factor	Item factor loading			Reliability indices	
		Coef	(SE)	Stand. Coef	Reliability coefficients	AVE [√(AVE)]
DMQ3	< – Social	1		0.826	α = 0.838; ω = 0.843	0.659 [0.812]
DMQ11	< – Social	1.067	(0.021)	0.882		
DMQ14	< – Social	1.082	(0.021)	0.894		
DMQ16	< – Social	0.743	(0.028)	0.614		
DMQ8	< – Conformity	1		0.849	α = 0.899; ω = 0.901	0.825 [0.908]
DMQ12	< – Conformity	1.036	(0.023)	0.879		
DMQ19	< – Conformity	1.139	(0.023)	0.967		
DMQ20	< – Conformity	1.101	(0.023)	0.934		
DMQ1	< – Coping	1		0.830	α = 0.866; ω = 0.869	0.773 [0.879]
DMQ4	< – Coping	1.047	(0.023)	0.868		
DMQ17	< – Coping	1.129	(0.025)	0.937		
DMQ7	< – Pleasant Feeling	1		0.913	α = 0.850; ω = 0.851	0.819 [0.905]
DMQ13	< – Pleasant Feeling	0.983	(0.018)	0.898		
**Factor variances and covariances:**						
Parameter		Coef	(SE)	Stand. Coef	(95% CI Stand. Coef.)	
var(Social)		0.683	(0.023)	1		
var(Conformity)		0.720	(0.031)	1		
var(Coping)		0.689	(0.024)	1		
var(Pleasant Feeling)		0.833	(0.021)	1		
cov(Social, Conformity)		0.483	(0.027)	0.689	(0.635–0.742)	
cov(Social, Coping)		0.352	(0.024)	0.514	(0.456–0.572)	
cov(Social, Pleasant Feeling)		0.586	(0.022)	0.777	(0.739–0.815)	
cov(Conformity, Coping)		0.456	(0.028)	0.647	(0.590–0.704)	
cov(Conformity, Pleasant Feeling)		0.562	(0.028)	0.726	(0.676–0.775)	
cov(Coping, Pleasant Feeling)		0.521	(0.024)	0.687	(0.640–0.735)	
**Model goodness of fit statistics:**						
N		960				
RMSEA^a)^ (90% CI)		0.068 (0.061–0.075)				
CFI^a)^		0.983				
TLI^a)^		0.978				
SRMR		0.041				

Past 12 months alcohol users, age 25–64 years only, Czech adult population sample (2022)

The model defined as latent ordinal response variables CFA, based on polychoric inter-item correlations with observed thresholds. Estimation method – diagonally-weighted least squares (DWLS), mean-and variance-corrected

*RMSEA* root mean squared error of approximation, *CFI* comparative fit index, *TLI* Tucker-Lewis index, *SRMR* standardised root mean squared residual

^a)^ The scaled version of model fit statistic (model χ^2^ scaling correction factor c = 0.534)

α – Cronbach's α coefficient; ω – McDonald's composite reliability; AVE – average variance extracted

The key result of the final CFA conducted on the reduced set of DMQ-R items is that, for the adult respondents aged 25–64, the hypothesised four-factor structure was supported. The latent ordinal response CFA model provided a very good fit to the data, with the desired values of RMSEA (90% CI) below 0.08, CFI and TLI well above 0.95, and SRMR below 0.05. Similarly, the reliability indices for the respective latent factors also indicated very good reliability estimates of the scales (Cronbach’s α and McDonald’s composite reliability – all well above 0.800). In terms of the discriminant validity of the extracted factors, the AVE’s square root values (√AVE) were all higher than any of the estimated inter-factor correlations; thus, supporting the discriminant validity of the latent factors.

Next to these desirable properties of the final four-factor CFA model, it must be, however, also emphasised that for the 4th factor, only two items were retained in the reduced set of the DMQ-R (DMQ7 and DMQ13). Given that the wording of these two items refers to specific *feelings* associated with alcohol use, this 4th latent factor was appropriately renamed as ‘Pleasant Feeling’ (rather than the originally hypothesised factor of Enhancement).

## Discussion

The aim of the present study was to examine the validity of the four-factor structure of the DMQ-R instrument [11] in a representative sample of the Czech general population. Although the tested factorial structure was not fully replicated in the younger group (15–24 years), the four-factor model corroborated by the final CFA in adults (25–64 years) corresponds relatively well with the structure reported in previous research [14, 31].

Most previous studies on drinking motives have focused on the populations of adolescents and young adults. These studies, conducted from a cross-national comparative perspective [32–34], confirmed the four-factor structure of the DMQ-R. Similar results were also found in some Central European countries [13, 35]. In our study, the most notable difference between the current findings and previous studies is the merging of the Social and Enhancement domains into one common factor, which was found in the younger group of 15–24 year olds. Our results therefore suggest that the hypothesised Enhancement items are more likely to correspond to a common Social factor in the younger group.

Most research tends to associate social motives with frequent moderate drinking and enhancement motives with heavy drinking [31, 36]. For instance, studies by Kuntsche et al. [14, 32] suggest that among younger individuals, motives to consume alcohol occur particularly at social gatherings, celebrations, or parties. It has also been shown that younger individuals are more receptive to their environment and sensitive to social rewards from the external environment [37]. In similar vein, two recent studies conducted among the Czech adolescents also found that socialising with peers and time spent with friends are factors closely related to the alcohol use [38, 39]. Thus, in the younger population, these findings may partially support the merging of the two respective domains into one common domain.

On the other hand, in the 25–64 age group, our final CFA model supported most of the hypothesised factor structures of the DMQ-R. Specifically, our 13-item shortened version of the questionnaire was found to act as a reliable tool for the measurement of Social, Coping and Conformity motives. However, for the measurement of the fourth factor, Enhancement, only two items were retained in the final model, referring rather to specific Feelings than Enhancement itself.

The DMQ-R was originally designed to explore the motivational structure of alcohol use in adolescents and young adults. Nevertheless, recently, the need for an instrument specifically designed for adults led D’Aquino et al. [40] to develop a new version of the DMQ-R for the adult population, referred to as DMQ-A. In the DMQ-A, the original domain of Enhancement is broken down into two sub-domains (‘Taste’ and a newly defined sub-domain of ‘Enhancement’). In this new sub-domain of enhancement, D’Aquino et al. [40] emphasise the importance of two items referring to ‘*Feeling(s)*’ associated with drinking alcohol; namely, because a) ‘*You like the feeling’*, and b) ‘*It gives you a pleasant feeling’*. In addition to these two items, a set of extra items are added to the new enhancement sub-domain of the DMQ-A, asking whether drinking alcohol is fun *(‘Because it is fun’) *and makes you happy *(‘Because it makes you happy’)*. The results of our study are in line with D’Aquino et al. [40] in terms of highlighting the extraction of the factor with the two items referring to the ‘*Feeling(s)*’. However, our three remaining items that were hypothesised to correspond to the original Enhancement domain (items DMQ 9, 10 and 18) were not included in the final CFA model for the adult group aged 25–64. As we used a standard DMQ-R questionnaire in our study, we can only point out these differences and possibly consider the DMQ-A for future research.

The study has some strengths and limitations that should also be discussed. Even though the current study is drawn from a highly representative sample of the Czech population, respecting gender structure, different age groups and regions of the country, one should keep in mind that the respondent’s self-reporting bias could have influenced the data to a certain extent. For respondents it might appear desirable to report results appealing to the researchers. In this regard, some specific groups of respondents might want to minimise their scoring on selected items that can be viewed negatively (e.g. items corresponding to the Coping factor might be viewed as indicative for risky patterns of alcohol use and might be therefore underreported by a respondent). Furthermore, recall bias might also influence the results since the respondents are asked about their drinking motives, which most likely occurred in the past, when several days, weeks or even months could have passed.

Despite the limitations, the study is based on a solid representative sample, surveyed using a rigorous methodology; thus, with a presumably high reliability of the collected data. In a similar vein, the self-reported nature of the data can also be justified as necessary because the participant is the one who can most objectively report his/her own personal drinking motives. Furthermore, as Del Boca & Darkes [41] point out, when issues of anonymity and confidentiality are addressed in a study, participants’ self-reported responses are considered reliable. We emphasise that in our study these ethical principles were carefully addressed throughout the data collection process.

In terms of the implications of the study, we highlight the following issues that should be considered for future research and practice. First, we acknowledge that our study only partially replicates the original factorial structure of the DMQ-R in the younger population (15–24 years). In this respect, future research should include a larger sample of young Czechs in order to verify the latent structures outlined in this study. The current sample (*n* = 163), yet not small, might not be enough to fully demonstrate the validity of a 20-item questionnaire, and therefore, a more robust sample size is needed. Second, the results found among the adults (25–64 years) suggest that the DMQ-R is a reliable tool for measuring the drinking motives in terms of Social, Coping and Conformity domains. In this respect, we thus recommend the application of the questionnaire for assessment of these three motivational factors among the Czech adults. Similarly, the DMQ-R could also be used in clinical settings to screen for health-risk motives for alcohol use, especially with regard to the endorsement of the Coping factor. However, the dimensionality of the fourth hypothesised factor of Enhancement, ‘Pleasant Feeling’ respectively, should be the subject of more detailed research in the future.

## Supplementary Information

Supplementary Material 1.

## Data Availability

Data relevant to all analyses performed in the study were either presented in tables or uploaded as supplementary material in aggregated form as pairwise correlation matrices. Microdata on individual responses of study participants were not provided.

## Abbreviations

AVE: Average variance extracted
CFA: Confirmatory factor analysis
CFI: Comparative fit index
CI: Confidence interval
DMQ: Drinking Motives Questionnaire
DMQ-A: Drinking Motives Questionnaire, revised version for adults
DMQ-R: Drinking Motives Questionnaire, revised version
DWLS: Diagonally-weighted least squares
EFA: Exploratory factor analysis
RMSEA: Root mean squared error of approximation
SRMR: Standardised root mean squared residual
TLI: Tucker-Lewis index

## Footnotes

1 The study considered participants of working age (15–64 years), thus excluding respondents of senior age. The prevalence of alcohol use is relatively low in the senior population and it could also be argued that the drinking motives are specific to this group. For the analysis of the DMQ-R we therefore restricted the upper age limit of our sample to the conventional limit of working age below 65 years.

2 Respecting a common convention, the following values of the CFA goodness of fit indices were desired: RMSEA (90% CI) < 0.08, CFI and TLI > 0.95, SRMR < 0.05.

3 The analytical approach based on the latent ordinal response CFA is particularly recommended when the ordinal scale of the response items is of limited range (typically, 5 response levels or less). The importance of an appropriate approach is even more pronounced when the items are characterised by a significant deviation from the multivariate normal distribution (in terms of item skewness and/or kurtosis). In addition to the limited scale range of the ordinal DMQ-R responses, significant deviations from normality were found in several of the input DMQ-R items, as further documented in the Results section. The application of the latent ordinal CFA framework, as defined by the seminal work of Muthén [21], was therefore warranted.
